# Cancer Incidence and Mortality in a Cohort of US Blood Donors: A 20-Year Study

**DOI:** 10.1155/2013/814842

**Published:** 2013-12-31

**Authors:** Farnaz Vahidnia, Nora V. Hirschler, Maria Agapova, Artina Chinn, Michael P. Busch, Brian Custer

**Affiliations:** ^1^Blood Systems Research Institute, San Francisco, CA 94118, USA; ^2^Blood Centers of the Pacific, San Francisco, CA 94118, USA; ^3^University of Washington, Seattle, WA 98195, USA; ^4^Laboratory Medicine, University of California, San Francisco, CA 94143, USA

## Abstract

Blood donors are considered one of the healthiest populations. This study describes the epidemiology of cancer in a cohort of blood donors up to 20 years after blood donation. Records from donors who participated in the Retroviral Epidemiology Donor Study (REDS, 1991–2002) at Blood Centers of the Pacific (BCP), San Francisco, were linked to the California Cancer Registry (CCR, 1991–2010). Standardized incidence ratios (SIR) were estimated using standard US 2000 population, and survival analysis used to compare all-cause mortality among donors and a random sample of nondonors with cancer from CCR. Of 55,158 eligible allogeneic blood donors followed-up for 863,902 person-years, 4,236 (7.7%) primary malignant cancers were diagnosed. SIR in donors was 1.59 (95% CI = 1.54,1.64). Donors had significantly lower mortality (adjusted HR = 0.70, 95% CI = 0.66–0.74) compared with nondonor cancer patients, except for respiratory system cancers (adjusted HR = 0.93, 95% CI = 0.82–1.05). Elevated cancer incidence among blood donors may reflect higher diagnosis rates due to health seeking behavior and cancer screening in donors. A “healthy donor effect” on mortality following cancer diagnosis was demonstrated. This population-based database and sample repository of blood donors with long-term monitoring of cancer incidence provides the opportunity for future analyses of genetic and other biomarkers of cancer.

## 1. Introduction

Blood donors are considered to be one of the healthiest populations due to donation eligibility requirements. Studies have suggested a lower incidence of cancer diagnosis and mortality in blood donors. Merk et al. and Edgren et al. estimated cancer incidence in Swedish donors and Swedish and Danish donors, respectively, and showed lower incidence of cancer—including hematological malignancies—in blood donors [1, 2]. These investigators also analyzed risk of cancer in longer-term blood donors relative to donation frequency and found no association between donation intensity and risk of cancer among blood donors [3].

We are unaware of any similar large-scale longitudinal study of cancer occurrence in the US blood donor population. Moreover, the Swedish and Danish studies did not retain samples from donors, in contrast to the existence of cryopreserved plasma and cellular sample repositories from a large number of US blood donors who consented to long-term outcome research studies in the past several decades. Establishment of a population-based database for long-term monitoring of cancer incidence and outcome among these US blood donors therefore provides a necessary base on which future analyses of genetic or other biomarkers of cancer can be conducted.

Here we describe characteristics of a sample of the California blood donor population who consented to collection and storage of repository samples of serum, plasma, or whole blood for future research. By linking identity of these donors with the California Cancer Registry (CCR) we identified donors who developed cancer after blood donation and were able to estimate incidence of cancer among donors for all cancers and by anatomic site. We also compared survival in blood donors with a primary cancer to a demographically matched sample of nondonor population with malignancies to investigate the so-called “healthy donor effect” on overall mortality.

## 2. Methods 

### 2.1. Retroviral Epidemiology Donor Study

Beginning as early as 1974, as part of blood transfusion safety studies in the United States the National Heart Lung and Blood Institute (NHLBI) and others funded the creation and maintenance of a series of large-scale blood specimen repositories [4]. These repositories of donor or linked donor and recipient specimens include three large repositories developed during the NHLBI Retroviral Epidemiology Donor Study (REDS) starting in 1991 that contain specimens from over 700,000 representative blood donors [4, 5]. Details of donor consent, blood collection, and sample processing methods have been previously described for each repository [6]. Briefly, REDS was a multicenter study awarded to 5 large, geographically dispersed community blood centers (Baltimore/Washington DC, Detroit, Los Angeles, San Francisco, and Oklahoma). Approximately 10% of these specimens were provided by consenting donors at a Northern California blood bank, Blood Centers of the Pacific based in San Francisco (BCP). At the time of donation, blood donors would not have had overt signs of illness because such signs would have excluded them from eligibility to donate. History of hematological cancer would have resulted in permanent deferral for blood donation, whereas donors with a history of other cancer types would have been eligible to donate blood one to three years after completion of treatment if they were in complete remission and free of symptoms, consistent with the American Association of Blood Banks (AABB) and FDA policies [7–9].

### 2.2. Data Linkage

We obtained electronic record files from the data coordinating center for REDS (Westat Inc., Rockville, MD) that included the repository specimen number, donor and donation identification numbers, and dates of donation(s) in the repositories for BCP blood donors who had consented to participate and donated specimens to any of the three REDS repositories between 1991 and 2002. The files were provided to BCP information technology (IT) staff who then submitted an electronic file containing personal identifiers of the REDS repository donors to CCR to be matched against their records between January 1991 and December 2010.

The CCR is a population-based registry that maintains records of nearly all cancer cases diagnosed in California [10]. Statewide cancer reporting has been mandatory since January 1, 1988. For this linkage, the CCR's January 2012 extract was used. The linked dataset is complete through 2009 for donors with a cancer record. CCR performed a formal data linkage and sent deidentified records containing the specimen number and demographic, socioeconomic, and cancer variables (cancer diagnosis, cancer biomarkers, cancer description, treatment, and outcome) to Blood Systems Research Institute (BSRI) investigators for data analysis. All donor records were returned to BSRI investigators and kept in the master database irrespective of successful linkage to CCR records.

The linked database was also matched against the Social Security Administration (SSA) Death Master File (DMF) to verify vital status of blood donors and date of death for those who are no longer alive. The SSA has been collecting information for DMF since 1936. The SSA-DMF contains over 89 million records of deaths. An encrypted file including social security number, first and last name, date of birth, and date of last visit to blood center was sent to the SSA and compared to the DMF to identify matches. We verified information received from SSA with CCR and BCP records, using multiple time points and data elements, including date of last visit to the blood center, cancer diagnosis date, hospital admission date, and last date known alive/dead in CCR and BCP records. Where there was a discrepancy among multiple sources, we assumed BCP records to be the gold standard with respect to vital status, because donors must be physically present at BCP for each donation visit; followed by CCR, which annually updates vital status of cancer patients using multiple national and local sources; and then SSA-DMF.

To compare overall survival of donor and non-donor cancer patients, we also requested a comparison group of frequency matched non-donor cancer records from CCR in a ratio of 1 donor with cancer to 3 nondonors with cancer (Figure 2). The linked BCP/CCR cohort database (*n* = 66, 984) was divided into one of 26,400 categories. The categories were determined by a unique combination of “year of diagnosis/quintile of SES/race/sex/SEER tumor categories” (22 years of birth certificates × 5 months × 6 race categories × 2 sex categories × 20 SEER tumor categories = 26,400 total categories). A uniform random number was assigned between zero and one to each of the non-donor birth certificates in CCR database. Then, the records were sorted in each of the 26,400 categories by this random number. Records in each category were selected for inclusion in the study dataset with the first record up to three times the number of matches in that category to achieve a 3 : 1 ratio of non-donor to donor records.

IRB approvals to conduct this data linkage and analysis were obtained from the University of California, San Francisco Committee on Human Research (the IRB of record for BSRI/BCP), and from the California Public Health Service (the IRB of record for CCR).

### 2.3. Master Database

The resulting linked master database contains demographic and donation history data from BCP's database for 66,984 donors and CCR records for the subset of these donors who were diagnosed with cancer between 1991 and 2009.

*BCP Data*. The BCP dataset consists of the demographic information required by the CCR in order to link donors to cancer registrants: first name, middle initial, last name, social security number, date of birth, sex, last known street address, city, and zip code. Donors' current address is actively updated only when donors visit the center seeking to donate. This dataset also contains the following demographic and donation history variables: birth state and/or country, race/ethnicity, marital status at time of last BCP visit, education, first or repeat status on repository specimen donation, donation type, date of repository specimen donation, first donation date, total number of presentations to attempt to donate, total number and types of successful donations, last donation date, and ABO/Rh blood groups [11]. 

*CCR Data. *The CCR dataset includes additional variables in addition to demographics related to the following domains for donors and nondonors who had a cancer:

1. socioeconomic: occupation, median household income, education index (average years of schooling in a given census tracts), college degree index, poverty index, socioeconomic status (SES) index, and urban/rural status;
2. cancer diagnosis: date of diagnosis, date of admission, primary or secondary tumor, number of tumors, tumor size, and diagnosis location;
3. cancer description: tumor primary site (SEER ICD-O-2 and ICD-O-3 definition), tumor stage at diagnosis, histologic type and grade/differentiation, and regional node extension;
4. cancer biomarkers (available for some records): estrogen receptor for breast cancer, acid phosphatase for prostate cancer, alphafetoprotein for testicular cancer, carcinoembryonic antigen (CEA) for colorectal cancer, and carbohydrate antigen (CA-125) for ovarian cancer;
5. treatment: type of chemotherapy, hormonal therapy, and immunotherapy;
6. outcomes: vital status, date of last contact/death, place of death, and cause of death.

### 2.4. Exclusion Criteria

**Figure 1:**
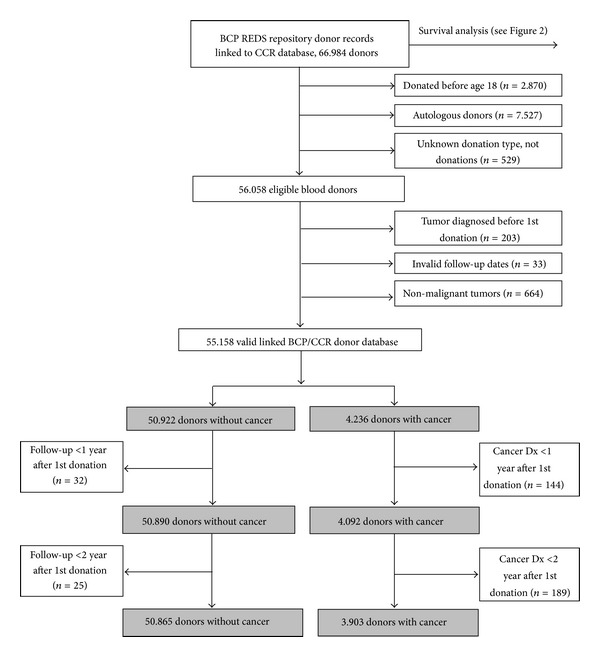
Flow diagram of master linked database, CCR: California Cancer Registry, BCP: Blood Centers of the Pacific, San Francisco, CA, 1991–2009. Note: eligible study populations analyzed to estimate standardized incidence ratios are shown in gray.

We excluded donors who donated before age of 18 (*n* = 2, 870), donors who gave autologous donations (*n* = 7, 527), donors with unknown donation type or confirmatory testing blood sample (*n* = 529), and those who were diagnosed with a cancer before their first donation to REDS repositories or had invalid date records in the linked database (*n* = 236) (Figure 1). We further excluded 664 donors who were diagnosed with a primary nonmalignant tumor (SEER “behavior” variable coded as benign, borderline, or in situ tumor) during study followup. We also excluded one year and two year periods following first donation from the analysis to ensure exclusion of donors with potentially undiagnosed cancer at time of donation or cancers diagnosed shortly after donation (*n* = 57 and 333, resp.).

**Figure 2:**
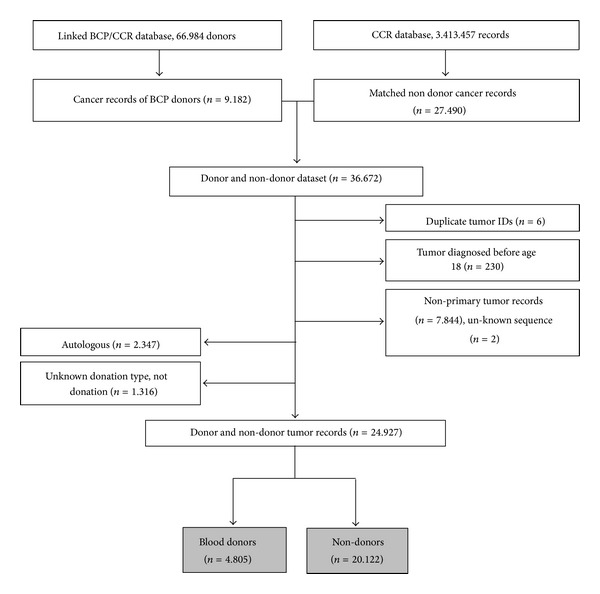
Flow diagram of donor and non-donor cancer records used for survival analysis, BCP: Blood Centers of the Pacific, CCR: California Cancer Registry, San Francisco, CA, 1991–2009. Note: study population for survival analysis is shown in gray.

For survival analysis (donors and nondonors with cancer) we excluded records of donors with autologous/unknown donation type (*n* = 3, 663), nonprimary tumors (*n* = 7, 844), tumors with unknown sequence (*n* = 2), duplicate tumor IDs (*n* = 6), or a tumor diagnosed before age of 18 (*n* = 230) (Figure 2).

### 2.5. Statistical Analysis

Number of diagnosed cancer cases for all cancers and by anatomic site were estimated using SEER ICD-O-3 site codes [12]. Donors' follow-up time and all-cause mortality were updated using “vital status” captured in CCR data defined as “patient's vital status as of the date of data extraction” (completed through 2009) [13]. For donors without a cancer record, last follow-up time was updated using matched SSA-DMF records. Donors were followed from first repository donation to cancer diagnosis, death, or the end of study followup (December 31, 2009), whichever came first.

The observed incidence rate of any cancer among BCP donors was compared with the rates expected based on SEER 9 registry data, including Atlanta, Connecticut, Detroit, Hawaii, Iowa, New Mexico, San Francisco-Oakland, Seattle-Puget Sound, and Utah. The number of expected cancer cases was calculated using multiplication of person-years of followup by the sex- and age-specific SEER incidence rates for the calendar years 1992 to 2009. We also estimated the expected cancer cases using only San Francisco-Oakland SEER data. Standardized incidence ratios (SIRs) were calculated by dividing the number of observed over expected cases for all cancers and by anatomic sites. Sensitivity analysis was conducted to estimate SIRs using different latency periods between first repository donation and primary cancer diagnosis and to exclude potential cancer patients that were not diagnosed at time of first donation (Figure 1). We estimated SIR for all eligible donors with a valid record (*n* = 55, 158); then we limited the eligible study population to donors who had more than one year of follow-up (*n* = 54, 982) and to those with two years or longer of follow-up time (*n* = 54, 768). Confidence intervals (CI) for the SIRs were calculated based on Poisson distribution as described in Rothman and Boice [14].

Hazard ratios (HRs) and 95% CI for death following cancer diagnoses were calculated using Cox proportional hazard regression models for all primary cancers combined and by cancer site, adjusted for age at diagnosis, sex, race, SES, tumor stage, and grade at diagnosis to compare all-cause mortality among donors and nondonors diagnosed with primary cancer (Figure 2).

All data analyses were performed using STATA 11.2 (STATA Corporation, TX, USA). All statistical tests were two sided with a 5% type I error.

## 3. Results 

A total of 66,984 BCP donors who had consented to one or more of the REDS repository studies were searched for a link to 3,413,457 CCR records. The linkage resulted in 7,943 individual donors with cancer. Between 1991 and 2009, 4,236 primary malignant cancer records were identified among 55,158 blood donors eligible for this analysis (Figure 1).

Demographic characteristics and donation history of eligible BCP blood donors with and without a primary cancer record are presented in Table 1. Compared to donors without cancer, donors who developed cancer were older at time of first repository donation and overrepresented white males and donors with lower education levels. Donors with a cancer record had a shorter follow-up time compared to donors without a cancer (median 9.8 years versus 16.9 years), a longer duration of donor activity (median: 3.7 years versus 3.1 years), and a greater number of lifetime blood donations (median 6 donations versus 4 donations).

### 3.1. Cancer Incidence among BCP Blood Donors

During 863,902 person-years of follow-up, 4,236 primary cancers were observed among BCP blood donors, while 2,670 cancers were expected (SIR = 1.59; 95% CI = 1.54–1.64). Excluding donors with less than one year of followup, the number of observed primary cancers decreased to 4,092 cases during 808,855 person-years of followup (Table 2). Restricting the analysis to exclude donors with less than one or two years of followup slightly increased the ratio of observed to expected cancers for about 5–10% (SIR = 1.59; 95% CI = 1.54–1.64 with no latency period, versus SIR = 1.64, 95% CI = 1.59–1.70 with one year latency period (Table 2) and 1.69, 95% CI = 1.64–1.74 with two years latency period). The most common sites of cancer diagnosed among BCP blood donors with more than one year of followup were male genital and breast cancers (*n* = 1, 000 and 754, resp.) followed by cancers of digestive system (*n* = 523). Compared with the general US population, BCP blood donors had a higher number of observed cases for most cancer sites than expected (Table 3). Using “San Francisco-Oakland SEER registry” incidence rates as a reference for the same time period slightly changed the SIRs for all cancer sites (data not shown); however, the changes in SIR using different reference populations were not substantial. The largest change was observed for female malignant skin cancers with a 27% increase in the estimated SIR when using San Francisco-Oakland SEER registry rates (SIR = 2.26, 95% CI = 1.88–2.68) as compared with Nine SEER registry rates (SIR = 1.77, 95% CI = 1.48–2.11).

### 3.2. Mortality following Cancer Diagnoses in Donors versus Nondonors

**Figure 3:**
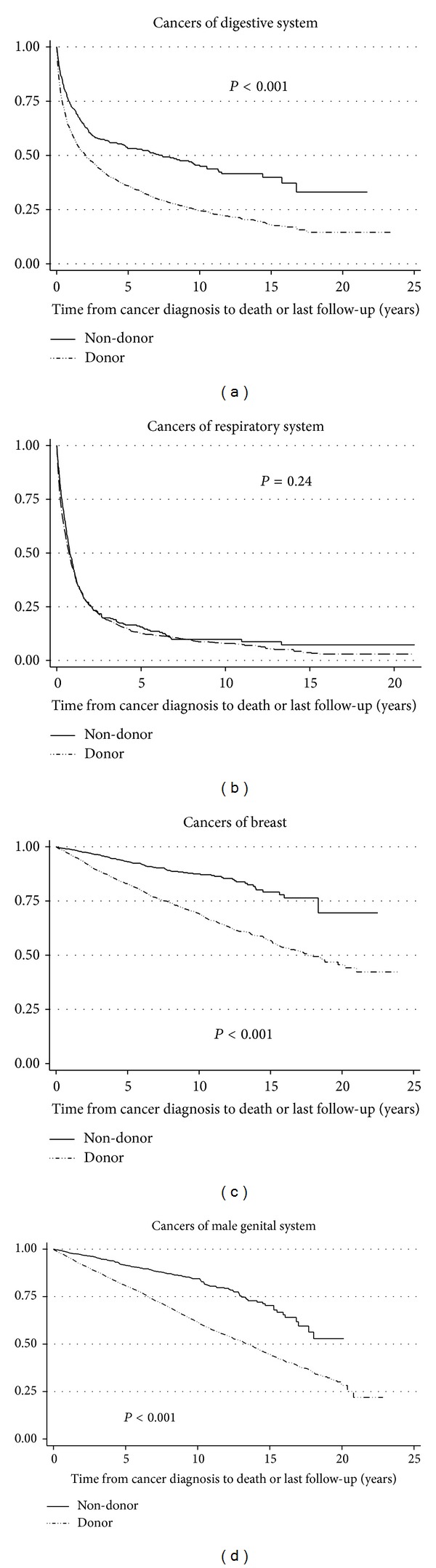
KM survival curves for all-cause mortality for donors and nondonors with (a) cancers of digestive system (*P* < 0.001); (b) cancers of respiratory system (*P* = 0.24); (c) breast cancers (*P* < 0.001); (d) cancers of male genital system (*P* < 0.001); *x*-axis represents survival time in years.

We compared BCP allogeneic blood donors with a primary cancer record (*n* = 4, 805) to a demographically matched sample of the California cancer population (nondonors, *n* = 20, 122). In analyses adjusted for cancer site, we found that donors had a significantly higher overall survival following diagnoses of cancer compared with nondonors regardless of the cancer site (*P* < 0.001, data not shown). In stratified analyses by cancer site, we observed a significantly higher overall survival among donors with site-specific cancers (*P* < 0.001, Figures 3(a), 3(c), and 3(d)), except for cancers of respiratory system (*P* = 0.24, Figure 3(b)), brain and nervous system, and myeloma (*P* = 0.09 and 0.96, resp., data not shown).

In multivariable Cox regression models, risk of mortality was significantly lower among donors compared with nondonors for most cancer sites after adjusting for age at diagnosis, sex, race, SES index, tumor stage, and grade at diagnosis, with an unadjusted HR of 0.58 (95% CI = 0.55–0.62) and an adjusted HR of 0.70 (95% CI = 0.66–0.74, Table 4). Risk of all-cause mortality among donors with cancers of the respiratory system and multiple myeloma remained nonsignificant in the adjusted Cox regression models.

## 4. Discussion

### 4.1. Standardized Incidence Ratio

The present study describes the epidemiology and incidence of cancer among a large cohort of US blood donors for up to 20 years after blood donation. We observed an increased incidence of cancer diagnoses among BCP blood donors as compared with general US population, as well as lower overall mortality among donors who were diagnosed with cancer compared with a matched non-donor cancer population.

Investigators with the Scandinavian Donations and Transfusions Database (SCANDAT) linked data on all registered blood donors and recipients in Sweden and Denmark with national population and health registries with complete followup up to 36 years [15]. The investigators reported lower mortality and cancer incidence among blood donors than among the general Swedish and Danish populations [1]. The current study design is similar to Scandinavian study in terms of linking donation database to health registries to estimate cancer incidence among blood donors. However, there are major differences between the two study designs. The most important one is the existing national health registers and nationwide transfusion registries in the Scandinavian countries, which capture almost all Swedish and Danish population. The “complete followup” of the donors in SCANDAT minimizes the likelihood of surveillance bias in the Scandinavian study, while our analysis is limited to BCP blood donors and cancer cases reported within California. Another major difference is the relatively small size of our study population with about 4,000 cancer cases, compared with 38,000 cancers recorded in the SCANDAT. However, the current analysis is the first attempt to report on long-term cancer outcome of blood donors in the US.

Likely explanations for increase in cancer incidence among BCP donors relative to the general US population are the differences in demographics and access to primary care in BCP blood donors and the general population. BCP blood donors are disproportionately young and white and are more educated than the general US population. The higher incidence rates of breast and male genital system cancers for which screening of healthy adults is available and recommended, for example, could be due to known differences in education level, race, SES, geographic location, or unmeasured differences in health-seeking behavior and access to health care for blood donors compared to the general US population. Our SIR estimates do not take into account the stage and grade of cancer at time of diagnosis (and hence entry into CCR database); a more educated donor with more frequent primary doctor visits and access to preventive care and screening is more likely to be diagnosed with a low grade cancer at earlier stages than the general population [16]. Interesting finding of the Scandinavian study is higher incidence of breast and prostate cancer in their donor population compared with the reference population [1].

### 4.2. Lower Mortality among Blood Donors versus Nondonors

Our study documented lower overall mortality following diagnoses when comparing blood donors with cancer and non-donor cancer populations. Selection bias due to “healthy worker effect” has been indicated in relation to various diseases including cancer [17–20]. However, there are no studies of a “healthy donor effect” on overall survival in blood donors with cancer. Lack of a significant dose-effect relationship between blood donation and overall mortality among BCP donors with cancer (data not shown) suggests that “healthy donor effect” on mortality is through pathways not related to duration of donor activity or number of lifetime donations. Many behavioral and medical factors related to cancer incidence and survival are not included in the blood donor screening and eligibility criteria. For instance, smoking history or alcohol consumption are known risk factors for cancer incidence and mortality [21, 22] but were not collected for REDS (or other US) donors, because smoking or drinking are not a reason for donor deferral. This could partially explain similar survival rates among donors and nondonors with primary cancers of the respiratory system.

Better survival among BCP blood donors with cancer after adjusting for multiple confounding factors suggests the possibility of differences in access to health care resulting in earlier diagnosis and better treatment of malignancies. One might again assume health seeking behavior of blood donors and lag time effect as an explanation, that is, more frequent doctor's office visits and screening during donor's lifetime; and therefore, cancers were diagnosed at earlier stages with a better prognosis [23]. This is only true for cancers that could be diagnosed during presymptomatic stages by the current recommended screening practices, that is, breast, prostate, and colorectal cancers. Nonetheless, selection bias due to overall healthier profile of donors, access to health care, and treatment options or “healthy registration effect” [23] are more plausible explanations, because the effect remains significant after adjusting for tumor stage and grade. Thus, the increased incidence of cancer and the better survival following cancer diagnoses are linked.

### 4.3. Study Limitations and Strengths

The present data is limited by differential ascertainment of the vital status for donors with and without cancer. For donors with a cancer record in CCR the “vital status” variable is updated through various registries and data sources (source of last followup information is the CCR) [13], with a complete followup through December 2009. However, vital status for donors without cancer was updated using only SSA-DMF records.

The SSA does not guarantee the accuracy of their SSA-DMF records. Thus, the absence of a particular person is not proof that this person is alive. Based on age-adjusted US mortality rates from 1997 to 2009 [24], we expected about 5,200 deaths among 64,000 blood donors within an average 10 years of followup. However, SSA-DMF search reported about 4,300 matched death records for our donor population (82%). We verified vital status of blood donors using multiple time points, including last visit to BCP to increase accuracy of our follow-up time. However, an erroneous classification of a deceased donor as being alive by the end of study followup is plausible, which will result in overestimation of person-years of followup, overestimation of expected cancer cases, and underestimation of SIR. On the other hand, we speculate that our observed cases are underestimated, due to ignoring primary tumors that may have been diagnosed outside of California after blood donation. Knowing the last visit date to the blood center, 15% of eligible donors without a cancer record in CCR visited BCP in 2008 or 2009, which we can consider correctly classified as cancer-free. For the other 85%, it is not feasible to verify California residency beyond their last blood donation date. We cannot ascertain if they were diagnosed with a primary tumor outside of California and if they had been diagnosed in California, the number of observed cases would have been larger, resulting in greater observed to expected ratio and larger SIR. The person-years of followup for these donors, on the other hand, would have been shorter, because they would have contributed person-time up to cancer diagnosis, not to the end of study (or date of death by SS-DMF). Smaller person-years of followup would have reduced the estimate for expected cancers and increased observed to expected ratio and SIR. Thus, we consider the overall bias in SIR estimates, if any, to be towards the null.

In another set of sensitivity analyses we calculated a range of SIRs without updated vital status information. We estimated SIRs assuming two extreme ranges for person-years of followup: from first donation to cancer diagnosis or December 31, 2009 (SIR = 1.52, 95% CI = 1.47–1.56) or from first donation to cancer diagnosis or last visit to the blood center (SIR = 3.47, 95% CI = 3.37–3.58). Our conservative estimate after vital status update and excluding donors with less than 12 months of followup time showed 64% greater incidence of primary malignant tumors among BCP REDS donors compared with the general US population (95% CI = 59–70%). While our data are observational with limited follow-up information for donors without a cancer record, sensitivity analyses assuming the best and worst case scenarios for donors without a cancer (all donors were alive until the end of study followup or all donors were lost to followup after last BCP visit, resp.) indicate that cancer incidence among our blood donor cohort exceeds that of the general US population in each extreme assumption scenario.

A second limitation of the current analysis is that we had access to limited demographic and medical history information for donors. Data on variables generally known as risk or prognostic factors for cancer, such as weight and height, smoking status, alcohol drinking, family history of cancer, and diabetes, are not captured in BCP or REDS donor data.

Third, our results are limited to northern California blood donors who participated in REDS during 1991–2002. Blood donors have different demographic and behavioral risk factor profiles than the general population, and furthermore BCP blood donors may be a group of blood donors that are different from the general US blood donor population in many aspects related to cancer incidence and mortality. Lastly, while non-donor cancer population is not reflected in BCP REDS, there is a possibility of misclassification if they donated blood at other times or other blood centers. If this is true, the actual donor, non-donor differences in mortality would likely be even greater than the current report.

This is the first report of cancer incidence among US blood donors in comparison with the general population. In the next phase of the study a larger dataset including additional REDS donor records from other participating blood centers will be matched against National Death Index to increase the precision and accuracy of the standardized cancer incidence and outcomes estimates among REDS blood donors. A noteworthy aspect of the existing linked donor-cancer database is the stored repository of whole blood and/or serum/plasma specimens which were collected years prior to cancer diagnosis. The current study establishes the capability of health outcomes linkage and presages utility of the stored specimens in a way that had not been considered during the design and creation of the REDS repositories. The establishment of the linked database for these repository donors is the first step towards further investigation of repository samples to investigate cancer biomarkers that may have been present in serum, plasma or whole blood samples during a time period when these individuals were healthy cancer-free blood donors. These investigations should help us to better understand biomarker development and changes that may be present in blood years before clinical cancer is diagnosed.

## Figures and Tables

**Table 1:** Characteristics of allogeneic donor study population with specimens in REDS repositories by cancer status—Blood Centers of the Pacific, 1991–2009 (*n* = 55,158).

	Cancer cases	Donors without a cancer
	*n* = 4,236 (7.7%)	*n* = 50,922 (92.3%)
First donation to repository (calendar year)		
Median (range)	1992 (1991–2002)	1993 (1991–2002)
Last known BCP visit (calendar year)		
Median (range)	1996 (1991–2009)	1997 (1991–2009)
Calendar year of cancer diagnosis		
Median (range)	2003 (1991–2009)	—
Sex, *n* (%)*		
Female	1,900 (44.9)	25,065 (49.2)
Age at first donation in years, *n* (%)*		
18–49	2,664 (62.9)	45,811 (90.0)
50–75	1,055 (24.9)	3,815 (7.5)
76 +	517 (12.2)	1,296 (2.6)
Mean (SD)	49.9 (12.3)	38.2 (11.9)
Race, *n* (%)*		
White	3,724 (87.9)	41,662 (81.8)
Education, *n* (%)^*∧*^		
College degree or higher	830 (19.6)	11,595 (22.8)
ABO blood group, *n* (%)		
Type O	2,039 (48.14)	24,341 (47.8)
Donation type		
Whole blood, RBC, *n* (%)	4,107 (97.0)	49,855 (97.9)
Duration of donor activity, *n* (%)**		
More than 3 years	2,279 (53.8)	25,504 (50.1)
Median (IQR)	3.7 (0.6–9.3)	3.1 (0.2–9.9)
Total number of blood donations, *n* (%)*		
>4 donations	2,329 (55.0)	24,207 (47.5)
Median (IQR)	6 (2–15)	4 (1–12)
Number of donated specimens to the repositories		
Median (IQR)	1 (1-2)	1 (1-2)
Study followup time in years*		
Median (IQR)	9.8 (5.4–13.7)	16.9 (15.6–18)

*P < 0.0001, **P < 0.001, ^*∧*^education information is missing for 60% of donors.

**Table 2:** Standardized incidence ratio (SIR) for all cancers, allogeneic blood donors with more than one year of followup, Blood Centers of the Pacific, 1991–2009 (*n* = 54,982).

Age group	Number of donors	Person-years	Observed	Expected	SIR	95% CI	
Males							
18–29	6,382	97,018.3	95	40.9	2.32	1.88	2.84
30–39	8,077	122,190.7	223	105.7	2.11	1.84	2.41
40–49	7,497	111,655.3	575	236.7	2.43	2.23	2.64
50–59	3,992	55,664.0	737	404.7	1.82	1.69	1.96
60–69	1,794	22,632.8	528	436.3	1.21	1.11	1.32
70+	349	3,928	102	121.2	0.84	0.69	1.02
Males total	**28,091**	**413,088.8**	**2,260**	**1,345.5**	**1.68**	**1.61**	**1.75**
Females							
18–29	7,546	112,935.3	160	57.4	2.79	2.37	3.26
30–39	7,894	118,319.1	346	162.3	2.13	1.91	2.37
40–49	6,609	97,661.8	561	336.5	1.67	1.53	1.81
50–59	3,275	45,759.0	444	314.5	1.41	1.28	1.55
60–69	1,356	18,481.9	275	226.5	1.21	1.07	1.37
70+	211	2,609	46	46.0	1.00	0.73	1.33
Females total	**26,891**	**395,766.1**	**1,832**	**1,143.2**	**1.60**	**1.53**	**1.68**
Total	**54,982**	**808,854.9**	**4,092**	**2,488.7**	**1.64**	**1.59**	**1.70**

Person-years of followup: from date of first repository donation to cancer diagnosis, death or Dec. 31, 2009.

SIR: observed/expected.

Expected: SEER age-adjusted and sex- and age-specific incidence rate × person-years of followup.

Age-adjusted incidence rates: SEER age-adjusted rates for malignant cases, years of diagnosis 1992–2009.

Standard population: US 2000 population.

**Table 3:** Standardized incidence ratio (SIR) by sex and cancer site, allogeneic blood donors with more than one year of followup, Blood Centers of the Pacific, 1991–2009 (*n* = 54,982).

	SIR	95% CI	
Males			
Oral cavity and pharynx	1.19	0.92	1.52
Digestive	1.24	1.10	1.38
Respiratory	0.93	0.79	1.08
Skin	2.19	1.89	2.53
Male genital	2.48	2.32	2.63
Urinary	1.15	0.97	1.37
Brain and nervous	1.55	1.11	2.10
Endocrine	0.78	0.47	1.21
Lymphoma	1.42	1.18	1.69
Myeloma	2.11	1.45	2.96
Leukemia	2.14	1.70	2.66
Other	1.45	1.16	1.78
Females			
Oral cavity and pharynx	1.01	0.60	1.60
Digestive	1.67	1.45	1.90
Respiratory	1.48	1.25	1.74
Skin	1.77	1.48	2.11
Breast	1.79	1.66	1.92
Female genital	1.50	1.32	1.70
Urinary	1.16	0.84	1.55
Brain and nervous	1.59	1.05	2.31
Endocrine	0.91	0.70	1.16
Lymphoma	1.72	1.39	2.12
Myeloma	1.79	1.02	2.90
Leukemia	1.33	0.88	1.92
Other	1.68	1.23	2.24

SIR was calculated using nine SEER registries age-adjusted, sex-, and age-specific incidence rates for years 1992–2009.

Other includes: mesothelioma, Kaposi sarcoma, eye & orbit, bone, soft tissue, miscellaneous (SEER site recode ICD-O-3 definition).

**Table 4:** Adjusted Cox proportional regression models for all-cause mortality, comparing donors and nondonors with cancer, by cancer site, Blood Centers of the Pacific and California Cancer Registry, 1991–2009 (*n* = 24,927).

	Donors			Nondonors			Adjusted HR*	P-value
	*n*	(%)	Mortality (%)	n	(%)	Mortality (%)		
All cancer sites**	4,805	(100)	27.1	20,122	(100)	45.0	0.70	<0.001
Followup < 5 years	2,519	(52.4)	42.0	11,319	(56.3)	59.1	0.75	<0.001
Followup ≥ 5 years	2,286	(47.6)	10.6	8,803	(43.7)	27.0	0.65	<0.001
By cancer site								
Oral cavity and pharynx	80	(1.7)	40.0	322	(1.6)	52.8	0.66	0.05
Digestive	559	(11.6)	48.1	2,395	(11.9)	68.9	0.70	<0.001
Respiratory	348	(7.2)	83.3	1,653	(8.2)	86.6	0.93	0.25
Skin	487	(10.1)	7.6	1,628	(8.1)	18.0	0.57	<0.01
Breast	914	(19.0)	10.5	3,451	(17.2)	27.2	0.51	<0.001
Female genital	405	(8.4)	16.8	1,735	(8.6)	32.1	0.68	<0.01
Male genital	1,025	(21.3)	14.2	5,010	(24.9)	39.3	0.57	<0.001
Urinary	241	(5.0)	30.7	1,078	(5.4)	53.4	0.70	<0.01
Brain and nervous	117	(2.4)	47.9	451	(2.2)	56.3	0.68	0.01
Endocrine	114	(2.4)	3.5	409	(2.0)	11.5	0.25	<0.01
Lymphoma	219	(4.6)	28.3	822	(4.6)	47.7	0.55	<0.001
Myeloma	50	(1.0)	66.0	176	(1.0)	68.2	1.00	1.00
Leukemia	104	(2.2)	51.0	385	(1.9)	65.2	0.70	0.03
Other^*∧*^	142	(3.0)	57.0	607	(3.0)	68.4	0.98	0.89

*Adjusted models include age at diagnosis, sex, race, tumor metastasis, tumor stage and grade, and SES.

**Cox models are stratified by cancer site; that is, baseline hazard is adjusted by site.

^*∧*^Other includes mesothelioma, Kaposi sarcoma, eye and orbit, bone, soft tissue, and miscellaneous (SEER site recode ICD-O-3 definition).
