# Erratum to “Cancer Incidence and Mortality in a Cohort of US Blood Donors: A 20-Year Study”

**DOI:** 10.1155/2014/301314

**Published:** 2014-07-24

**Authors:** Farnaz Vahidnia, Nora V. Hirschler, Maria Agapova, Artina Chinn, Michael P. Busch, Brian Custer

**Affiliations:** ^1^Blood Systems Research Institute, San Francisco, CA 94118, USA; ^2^Blood Centers of the Pacific, San Francisco, CA 94118, USA; ^3^University of Washington, Seattle, WA 98195, USA; ^4^Laboratory Medicine, University of California, San Francisco, CA 94143, USA

In the original paper, there was an error in Figure 3. In the legend for Figure 3, donor and nondonor lines were reversed. Here, we provide Figure 3 with the correct legend.

## Figures and Tables

**Figure 3:**
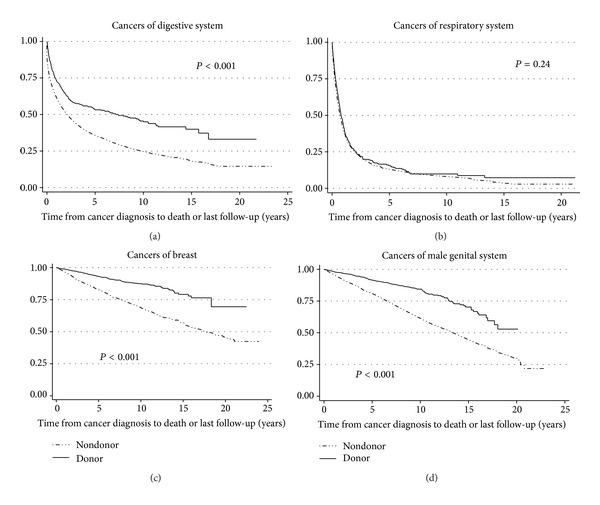
KM survival curves for all-cause mortality for donors and nondonors with (a) cancers of digestive system (*P* = 0.24); (b) cancers of respiratory system (*P* < 0.001); (c) breast cancers (*P* < 0.001); (d) cancers of male genital system (*P* < 0.001); *x*-axis represents survival time in years.

